# A Mechanical Model of Cell Segregation Driven by Differential Adhesion

**DOI:** 10.1371/journal.pone.0043226

**Published:** 2012-08-29

**Authors:** William R. Taylor, Rosalind Morley, Alexey Krasavin, Lauren Gregory, David G. Wilkinson, Alexei Poliakov

**Affiliations:** 1 Division of Mathematical Biology and Division of Developmental Neurobiology, MRC National Institute for Medical Research, London, United Kingdom; 2 Department of Physics, King's College London, London, United Kingdom; Aix-Marseille University, France

## Abstract

From simulations that begin with a random mix of two cell types, we monitor progress towards segregation driven by contact-mediated linkage of model cells, which is equivalent to the cell-cell adhesion of real cells. In comparison with real cell experiments, we show that this mechanical model can account for the observed extent of segregation obtained by differential adhesion in a 2D cell culture assay of cells with differentially expressed cadherin molecules. Calibration of virtual to real time allowed us to estimate a time course for these experiments that was within 50% agreement for the simulations compared to differential adhesion of cells. In contrast, simulations of differential adhesion do not account for the rate of segregation driven by interactions between EphB2 receptor and ephrinB1 expressing cells which occurs an order of magnitude faster. The latter result suggests that mechanisms additional or alternative to differential adhesion contribute to Eph-ephrin mediated cell segregation.

## Introduction

The generation of organised tissues during development requires that cell populations with distinct identity form discrete domains. This results in the formation of borders between tissues or regions, despite the potential for extensive intermingling due to cell intercalation during tissue growth, and the intrinsic motility of some cell types. Commonly, such borders are initially imprecise, with cells of distinct identity locally intermingled, and progressively become sharpened to form a flat interface. One key mechanism by which border formation is achieved is through the segregation of the cell populations from each other, and concomitant restriction of intermingling across the interface. There is thus much interest in the molecular and cellular basis of cell segregation.

An important approach comes from experiments in which cells from different tissues are dispersed then reaggregated in vitro to form a mixture, which is found to lead to segregation of the distinct cell types into discrete clusters [1]. By using such assays with cell lines expressing cadherins, which mediate cell-cell adhesion, it has been shown that cell segregation can be driven by differential affinity, due either to expression of different cadherins or different levels of the same cadherin [2], [3], [4]. The results of these studies have confirmed the differential adhesion hypothesis, in which segregation is proposed to be driven by the free energy of cell cohesion, which is lowest when contact is maximal between cells with the highest mutual affinity [5]. This requires that cells can move to interact with new neighbours, and it is proposed that this is due to a liquid-like behaviour in which they have intrinsic random motility.

There is extensive experimental support for the ability of differential adhesion to drive cell segregation in vitro, and which shows that cadherins contribute to tissue organisation in vivo [2], [3], [6]. However, it is unclear whether differential adhesion with random cell motility is sufficient to explain the rate or extent of cell segregation that can occur when distinct cell populations are mixed. Furthermore, there is increasing evidence for other mechanisms that can also drive segregation, including cell cortex tension generated by actomyosin contraction [7], and Eph receptor and ephrin signalling that can decrease cell-cell adhesion [8], [9] and mediate repulsion through regulation of the actin cytoskeleton [10], [11]. To better understand how cell segregation is achieved, it is important to determine whether each of the proposed mechanisms is sufficient in principle to account for the extent of cell segregation that is observed after mixing of cells.

In order to address the potential contribution of individual mechanisms to cell segregation, we have developed a computer model that simulates the adhesive and migratory behaviour of cells. This approach differs from the more commonly used Potts model of cell interaction which is based on a summation of surface interface energies [12]. At high cell density where there is little cell motion, the number of adhesive links formed between adjacent cells in our model will be equivalent to a packing energy, whereas at low density, the cells in our model can move more freely. This means that at low density we are able to calibrate the virtual time step in our simulations based on the rate of cell movement which allows us to not only see if segregation can occur but also to tell whether its rate matches that for real cells.

Here, we apply this computer model of adhesive interactions and compare it with cadherin-mediated and Eph-receptor/ephrin-mediated segregation in a 2D cell culture assay. We find that differences in adhesive interactions are in principle able to account for the extent and rate of segregation mediated by cadherins. However, for EphB2/ephrinB1-mediated segregation modelled as differential adhesion, the rate of segregation was slower by an order-of-magnitude compared with experimental observations, suggesting that other mechanisms contribute to or are responsible for segregation.

## Results

Using the model of cell motion and adhesion described in the Methods section, we carried out a number of simulation experiments to firstly characterise and optimise the model, followed by additional simulations which replicate the conditions used in experiments with real (living) cells. This comparison allowed us to establish an absolute time frame for the simulations. From this, the degree of segregation was compared to that observed in real cells over the same period. In summary, the simulations described below progress in the following sequence (with the corresponding Section number in parentheses):

- Using two equal sized populations of simulated cells;
  - Optimise the parameter unhook for segregation (2.1.1).
  - Measure segregation, varying runtime and density (2.1.2).
  - Test segregation over long simulation times (2.1.3).
- Given observed (real) cell segregation times then;
  - Using the results of Ab, map simulation to real time (2.2.1)
  - From Ba, get the time to attain observed segregation (2.2.2)
- Modifications to the model
  - Increase adhesion with contact time (2.3.1)
  - Increase cell steric exclusion (2.3.2)
  - Combination of Ca and Cb (2.3.3)

### 2.1 Simulations with a basic model

#### 2.1.1 Optimising cell “stickiness” for segregation

**Figure 1:**
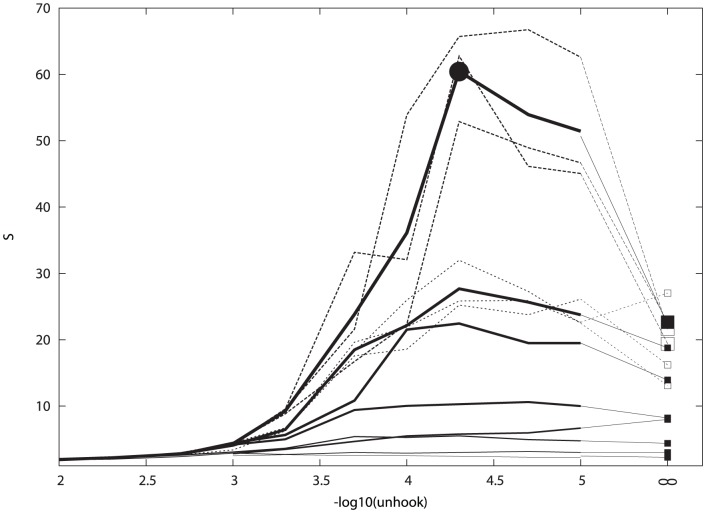
The effect of “stickiness” on segregation was measured by plotting the ratio of like-contacts and mixed contacts (S in **Equ. 2** on the Y-axis) against the value of the parameter unhook which controls the degree of cell cross-linking (X-axis, as negative log-value. i.e. 3 = 1/1000). The segregation score is plotted for simulations of different lengths from 1000 to 500 K steps, which appear as a series of traces with the longer runs always more segregated (higher). For the two longest runs (100 K and 500 K steps) the bold trace is the average over three runs shown by dashed lines. The results for unhook = 0 (i.e. no release) are plotted as squares at an arbitrary point to the right (marked by the infinity symbol) and connected to each trace by a dotted line for clarity. The large dot marks the maximum degree of segregation obtained with unhook (plotted at 4.3).

Given a fixed number of cells and the basic parameters of motion developed in our earlier work [13], the only aspects of the simulation that affect how much segregation occurs are the run time (‘virtual time’ or number of steps) and the “stickiness” of the cells. The latter property is determined by the decay constant on the lifetime of the inter-cellular links (‘hooks’) which is set by the value of the parameter unhook (see Methods Section. 4.1.2). In an analysis of cells at low density (100/field), the effects of stickiness only became apparent in the Mean Square Displacement (MSD) plots for values of unhook under. As this earlier analysis was for relatively short runs at low density with a single cell type, the full range of unhook was investigated again with run times from 1,000 up to 100,000 for two cell types (nominally, red and green) with adhesion only between cells of like type (Figure 1).

It can be seen in Figure 1 that for all except the shortest run lengths (where segregation does not have time to develop) there is a sharp transition from no segregation (score value of 2) for unhook values under 0.002 into segregating behaviour for values above this. As run length increases, so does the degree of segregation up to 500,000 steps, above which any additional improvement is slight. For these longer runs, the variation in the peak-ratio score (Equation 2) increases, partly because the denominator (the number of mixed contacts) is small in the score calculation but also because the chance configuration of a small number of large clusters can alter the score (see Figure 1b,c). To reduce this effect, the longest runs (100 K and 500 K steps) were repeated three times and an average taken.

The best segregation was found when the unhook parameter was in the range 10^−4^–10^−5^. The observation of a peak in this parameter range (between 4 and 5 in Figure 1) might result from the balance between two opposing trends: on one hand, the cells need to remain relatively fluid-like to allow the reassortment and fusion of scattered clusters, but on the other hand, strong links are needed to hold large clusters together once they have formed.

#### 2.1.2 Variation of cell segregation with run-time and density

**Figure 2:**
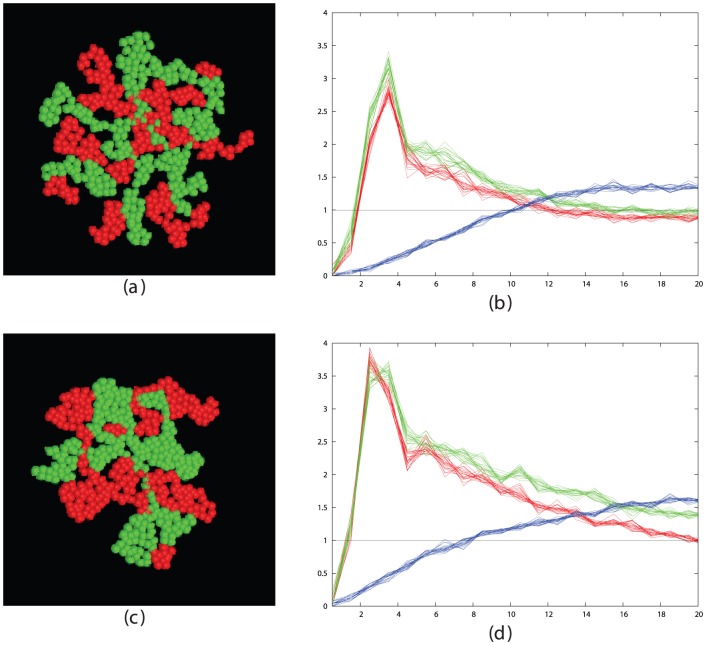
Simulated cell segregation is shown qualitatively (left panels) and quantitatively (right panels) as measured by the RDFs of the like cell types (red and green traces) and mixed cell types (blue traces). The RDF plots were calculated on a series of 10 equi-spaced frames over the final 1000 time-steps of the simulation. Parts *a* and *b* show the configuration after 50 K steps while parts *c* and *d* are after 500 K steps. The value plotted on the Y-axis is the peak-ratio score S = (R+G)/B, where R = red, G = green and B = blue peak areas summed between 2 and 4 on the X-axis. (See Methods section and Equation 2). The X-axis is marked in tens of microns (i.e. 3 = 30, which is the cell diameter in the simulations).

Taking the optimal values for cell-stickiness established in the previous section, the remaining factors affecting segregation are the cell density and the time the cells are allowed to move. To investigate this, we ran simulations for increasing times at different densities and monitored the state of segregation using the like∶mixed peak size ratio based on their radial distribution function (RDF). (See Methods section and Figure 2).

**Figure 3:**
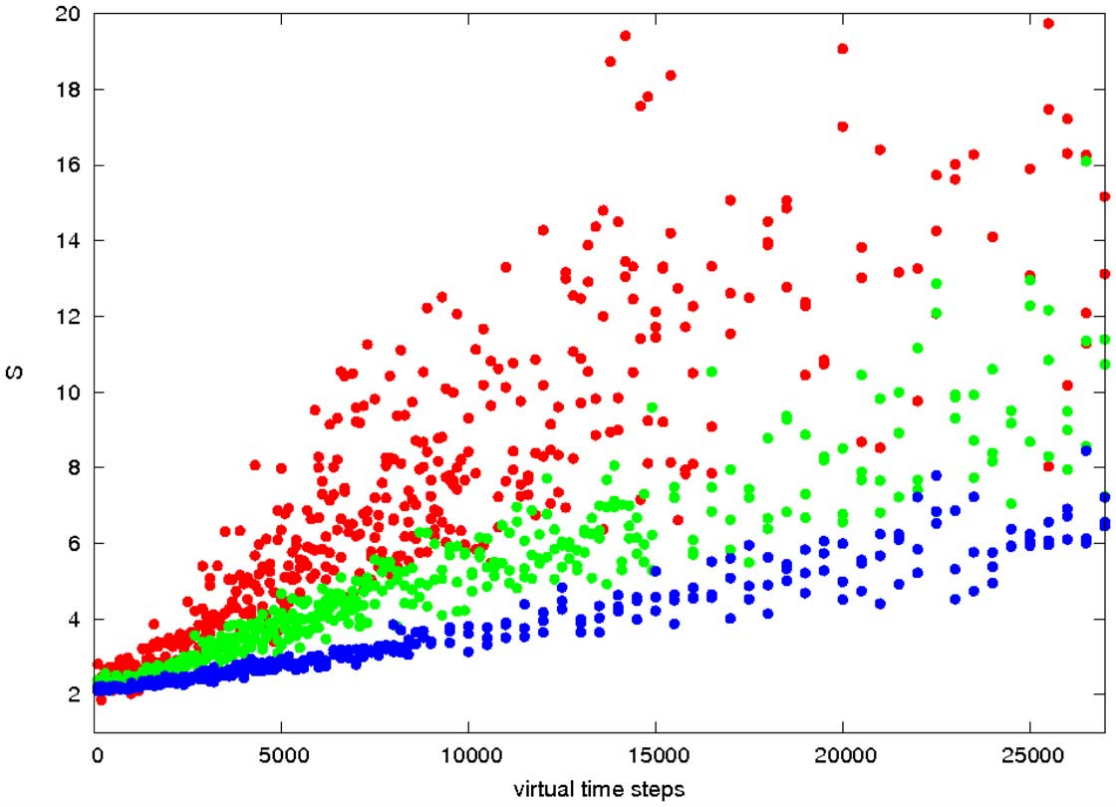
Relationship between segregation and cell density. The degree of cell segregation (**S** score, Y-axis) is plotted against simulation times as the number of time-steps (X-axis) for three different cell densities: red = low (200 cells/field), green = medium (500 cells/field) and blue = high (1000 cells/field). The latter is the maximum density for the circular field with radius 50 microns. The data were obtained from multiple simulations with unhook values of 0.0002, 0.0001 and 0.00005 across the optimal range.

From the plots and pictures described in Figure 2 and the examples in the Methods section, it is clear that segregation of cells into clusters of like-type can be obtained across the full range of cell density. To investigate the interrelationship of density and time, we took three cell densities corresponding to a low, medium and high density of 200, 500 and 1000 cells per field, with the latter being close to the maximum possible for close-packed cells and ran these over a series of run times, plotting the segregation peak-ratio score (**S**). (Figure 3). As would be expected, the denser cells took longer to attain the same degree of segregation and the less dense cells exhibit greater variation in score.

[n.b. The maximum density of the model can be calculated from the cell radius (*r*) of 15 microns and cells are confined within a field with radius (*R*) 500 microns. For hexagonaly (cubic) close packed cells, the length of the side (*t*) of the hexagon that contains a cell is: *t* = 2*r*/3^1/2^ giving the area (*a*) of the hexagon as: a = 3^3/2^/2t^2^ = (3^3/2^/2)(4r^2^/3) = 2r^2^3^1/2^. The area of the field, A = πR^2^ giving the maximum number of cells in the field (*N*) as: *N* = *A*/*a* = 1007. Given that some cells on the edge cannot be divided, 1000 is a reasonable approximation.]

Many of the clusters form extended interdigitating networks of red and green areas (e.g.: Figure 2) but none have attained a complete segregation of cells into one red and one green cluster. However, the trend for all densities remained towards fuller segregation over the period of the simulation.

#### 2.1.3 Segregation over long run-times

It was of interest to see if the linear trend towards more complete segregation seen in Figure 3 would continue or whether a stable state of segregation with multiple clusters would be attained. As the simulations that generated the results in the previous section were not particularly long the medium density model of 500 cells (250 red, 250 green) was selected and run over a much longer time course.

**Figure 4:**
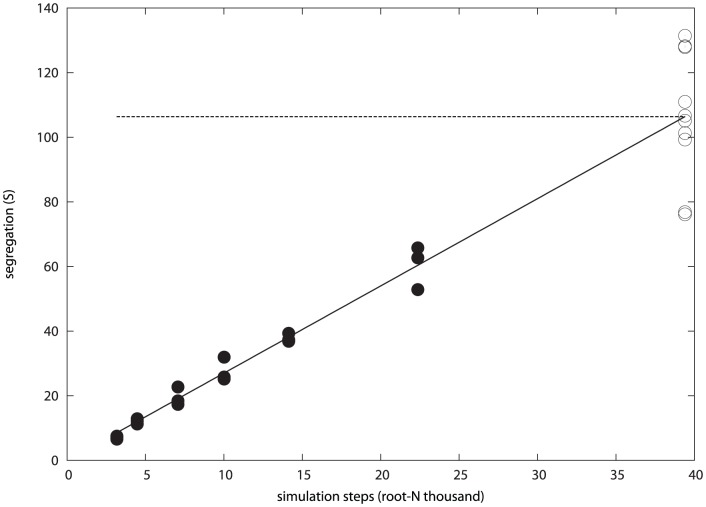
Cell segregation with simulation length. The degree of cell segregation as measured by the like∶mixed type ratio score (S, Y-axis) is plotted against ‘virtual time’ measured by the square-root of the number of time-steps in thousands (X-axis. e.g., 30 = 30×30 thousand = 900,000 steps). The filled circles are data from full simulations starting with a random mix of cell types and the open circles started from artificially segregated cells. The latter are plotted at the predicted time when the best-fit line to the full simulation data (solid line) cuts the mean value of the segregated data (horizontal dashed line).

The optimal value of unhook (0.00005) was used in a series of runs of increasing length over which it was found that with longer simulations, better segregation was still obtained as measured by the segregation score, but this was gained at an ever-diminishing rate. Fitting a function of the form ax^b^+c to these data found a value for *b* of 0.456 (+/−0.077) which was a reasonable approximation to a square-root. Plotting segregation against the square-root of the run-length revealed a linear relationship: 2.732 n^1/2^-0.433 or, more simply, re-fitted as 2.7 n^1/2^ (Figure 4).

**Figure 5:**
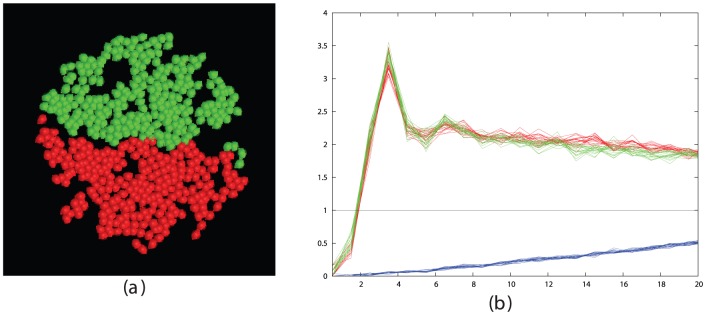
Artificial complete cell segregation. *a*, The configuration of red and green cells after 10 K steps, having started with the cells artificially split into a red and green half. *b*, The corresponding RDF (as in Figure 2).

To estimate how long it might take to attain full segregation, the cells were artificially separated at the start then allowed to equilibrate and cluster for 10,000 steps. The resulting configuration (repeated ten times) indicated what might be found after a very long run and was compared to the configurations found for a series of simulations with run times in the range of 10 K to 500 K steps (Figure 5). The relationship established in Figure 4 was then extrapolated to find the time at which full segregation might be attained as estimated by the artificially segregated populations. The intercept of this line with the mean value of the segregation score from the artificial splits (106.4) occurred after 1.5 M steps.

**Figure 6:**
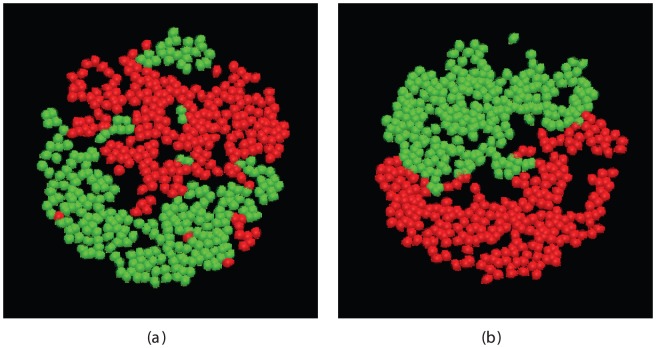
Full cell segregation is in panel *a* almost obtained and in *b* effectively obtained after simulations of five million steps.

The simulation runs at a million steps per hour, allowing some trial runs over the period estimated for full segregation. Several simulations of 1.5 M steps resulted in configurations similar to those of Figure 2 (after 500 K steps) but none had attained full segregation. Increasing the simulation length to 5 M steps, however, did lead to effectively complete segregation (Figure 6).

### 2.2 Comparison with real cells

The simulations described above were framed in terms of the virtual time that is set by the unit time-step of the simulation. This can be related to the time frame of real cells through comparison with data captured using time-lapse microscopy of cells and measuring their degree of segregation (Methods section).

For these studies, two types of cell were used. First, we used cells in which differential adhesion is mediated by the expression of two different cadherins (E and N); these cadherins have previously been shown to drive cell segregation [14], [15] and we have verified this in 3D hanging drop assays (data not shown). Second, we used cell lines in which differential expression of EphB2 receptor and ephrinB1 leads to cell segregation (See Methods and Ref.16). Eph/ephrin activation can promote differential adhesion [8], [9] and regulate cell migration [11], [16], but the contribution of these responses to segregation is not known. The comparisons thus enable us to address two questions: first whether the simulations match the results of differential adhesion-driven cell segregation, and second whether adhesive mechanisms alone can account for the extent and rate of Eph/ephrin-driven segregation.

**Figure 7:**
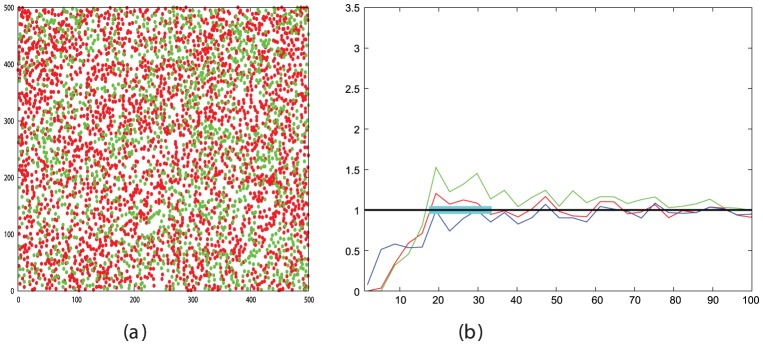
Cadherin mediated cell segregation. Frame *a* shows the digitised cell positions at the end of the experiment with the corresponding RDF plotted in part *b*, coloured as in previous RDF plots (See legend to Figure 2). The black line on the RDF plots is the expected random value and the light blue bar marks the region over which the peaks were summed to calculate the peak-ratio segregation score (S).

**Figure 8:**
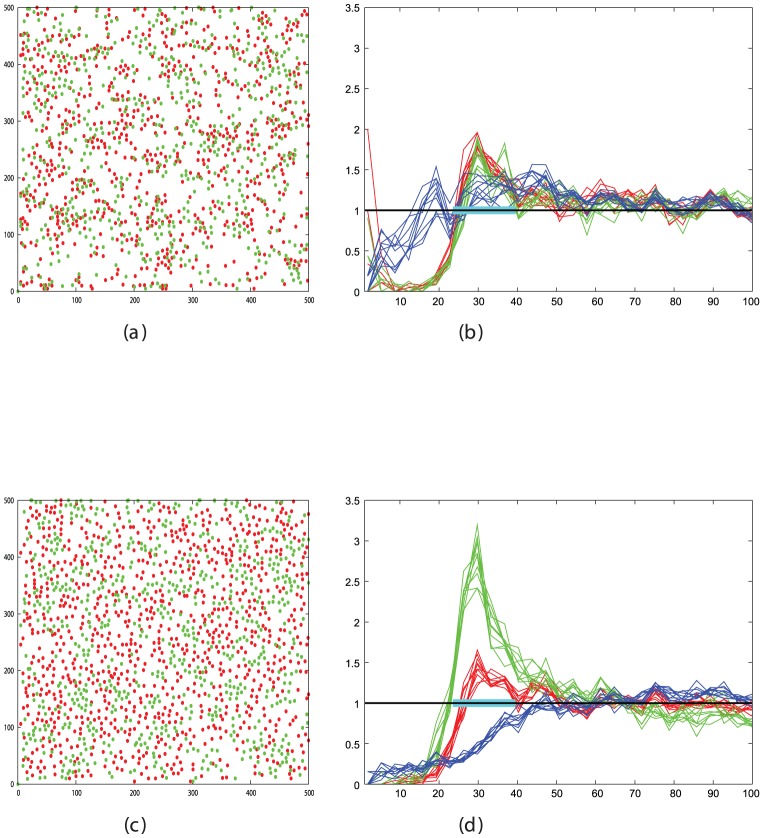
Eph/ephrin regulated real cell segregation. Panels *a*+*b* show the cell configuration and RDF near the start of the experiment plotted as in Figure 7 while panels *c*+*d* show the final state after 5 hours. The cell density is half that of the cadherin cells.

The cadherin expressing cells were close to a maximum simulation density of 950 cells/field and exhibited a weak degree of segregation even after the long time period of two days (Figures 7a, 7b). The Eph/ephrin expressing cells formed a medium density monolayer corresponding to a simulation density of 590 cells/field, which starting from a random configuration, as measured by the RDF analysis (Figures 8a, 8b) developed clear segregation after five hours (Figures 8c, 8d)

#### 2.2.1 Establishing an absolute timeframe

**Figure 9:**
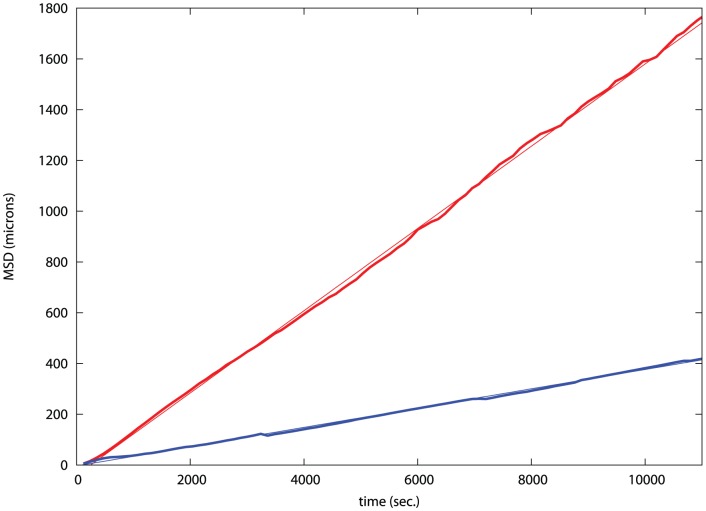
MSD plot for cell populations. The Mean square displacement, MSD (Y-axis, square microns), is plotted against time (X-axis, seconds) for the two cell lines: red, in which the EphB2 and ephrinB1 were expressed and blue for cells with differential expression of E and N cadherins. The fine lines are linear fits to the data with slopes: red = 0.16 and blue = 0.04 (the latter is such a close fit that it is almost invisible).

The diffusion of each cell group was estimated, prior to significant adhesion, by plotting their mean square displacement (MSD) against time, using the analysed trajectories of cell populations recorded by time-lapse microscopy (Figure 9). The plots for both cell populations have a good linear fit in their MSD plot which is characteristic of a random walk model for cell movement (Figure 9). The slower cadherin cells diffuse at 40% of the rate of the EphB2/ephrinB1 cells.

However, as we demonstrated previously [13], the slope of the MSD plot depends on the density of the cells (effectively a change in diffusion coefficient) and this was allowed for by running simulations for each observed density with a fixed intrinsic cell velocity and comparing this calculated MSD slope with the observed MSD slope. The observed∶calculated MSD ratios for each density were 0.038∶0.055 (N−/E− cadherin), and 0.16∶0.040 (EphB2/ephrinB1), giving rounded scaling factors of 0.7 and 4.0 for each group, respectively.

The distance scale in the simulations is 1 unit equals 10 microns, so when plotted as a squared value on an MSD plot, this is a factor of 100 relative to the observed plots. For the case of the EphB2/ephrinB1 cells where the ratio of the slope of calculated MSD plot is 4 times the observed (*F* = 4), the real-time (T_R_) to virtual-time (T_V_) ratio: T_R_/T_V_ = 100/F = 25. Similarly, for the simulated cadherin cells, *F* = 0.7, giving a ratio: T_R_/T_V_ = 145. [n.b. If *d* is the simulated displacement and *D* is the observed displacement (plotted as a squared value on the MSD plots), then the MSD slopes are S_V_ = d^2^/T_V_ and S_R_ = D^2^/T_R_ for the simulation (virtual) and observed (real) data, respectively, with T_V_ and T_R_ being the corresponding times. Since *D* = 10*d*, S_V_T_V_ = d^2^ = S_R_T_R_/100. Setting the scale factor F = S_R_/S_V_, then T_R_ = 100T_V_S_V_/S_R_ = 100T_V_/F and T_V_ = T_R_F/100.] Applying these factors for the conversion of the virtual time to true time allows the axis of Figure 3 to be read in real-time for each experiment.

#### 2.2.2 Comparison of observed and expected segregation

The cadherin cells had the lesser degree of segregation with a peak-ratio score, (Equation 2) of 2.8, with the EphB2/ephrinB1 cells having a clearer segregation with a score of 4.5. In principle, these levels can be used with the data for the appropriate density in Figure 3 to directly read off the virtual-time needed to reach each degree of segregation, which can then be converted to real-time using the scaling factors determined from the MSD plots.

**Figure 10:**
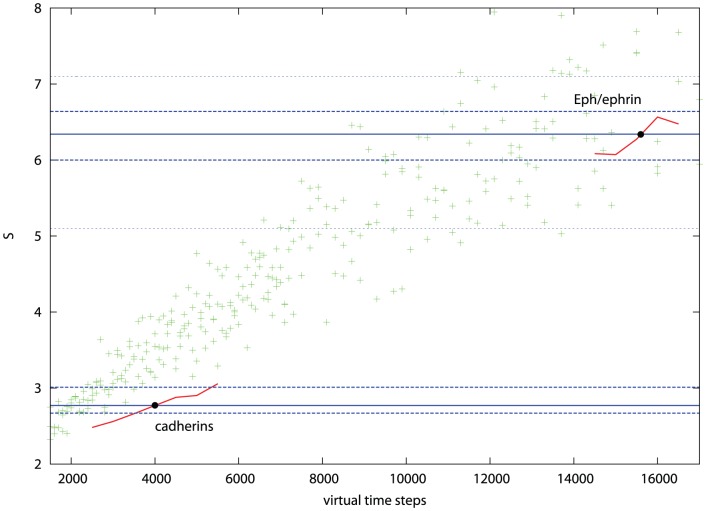
Simulated cell segregation at the density of real cells. Bottom left: high-density cells (950/field) simulated over the time interval 2500–5500 spanning the observed degree of segregation mediated by cadherins (lower solid blue line). Top right: medium-density cells (590/field) simulated over the interval (14500–16500) spanning the observed segregation seen for Eph/ephrin cells (upper solid blue line). The intersect of the simulated data (red) and observed values (blue) is marked by a black dot at 4000 and 15500 for the cadherin and Eph/ephrin cell lines, respectively. The dashed blue lines represent confidence intervals for the observed data (discussed below). The data for 500 cells/field from Figure 3 is plotted for reference (green crosses).

However, it will be apparent that the exact required cell densities are not plotted on Figure 3 and that there is considerable scatter, especially for the less dense cells. To improve the accuracy of this analysis, Figure 3 was used only to estimate a limited range in which the required point might lie for each cell group and a series of simulations was run over this range at the required cell density with each measurement being repeated 10 times to reduce noise. (Figure 10). The intercept of the mean peak-ratio score in each range with the observed value provided a better estimate for the virtual time needed to attain the observed degree of segregation.

For the cadherins and Eph/ephrins, these times were: 4000 and 16000, respectively (the latter approximating the plotted value of 15500) which when converted to real times using the MSD based factors derived in Section 2.2.1, gives: 4000×145 = 580 Ksec and 16000×25 = 400 Ksec, or 161 and 109 hours. The actual time courses of the experiments were 48 and 5 hours, respectively.

It is clear that the calculated times for both segregation experiments are greater than the observed times: by a factor of 3 for the cadherins and over 20 for the Eph/ephrins. There is little scope in the model to correct for these differences as most aspects are constrained: any change in the cell velocities will be normalised out by a corresponding change in the MSD plot slope and the “stickiness” of the cells is already optimised to induce segregation as quickly as possible. Before assessing alternative models, however, an analysis of possible errors was made to determine if the times, at least for the cadherins, might fall within the experimental range of variation.

#### 2.2.3 Analysis of errors

The aspect of the analysis that is most sensitive to noise is the segregation score which is based on a ratio of like∶mixed peak heights. With segregation, the size of the mixed peak (measuring the proximity of cells of differing types) will be small and more prone to error. To estimate this effect, the field of cells was split into left and right halves and top and bottom halves, with cell numbers being maintained by reflection. In addition, for the Eph/ephrin cells, the final ten frames of the experiment were available, although these will be partially correlated.

The segregation score measured for the cadherins ranged from 2.67 to 3.02 (plotted on Figure 10 as dashed lines), allowing at best only a slight reduction in the minimum time to 3000 time steps. This corresponds to a 50% reduction in real-time to 120 hours, compared to the actual time of 48 hours. For the Eph/ephrin cells, using the minimum observed score over the final 10 frames makes little impact, however, taking the minimum of the symmetry generated variations again reduces the simulation time by almost 50% to 70 hours but this is still more than an order of magnitude longer than the observed time.

**Figure 11:**
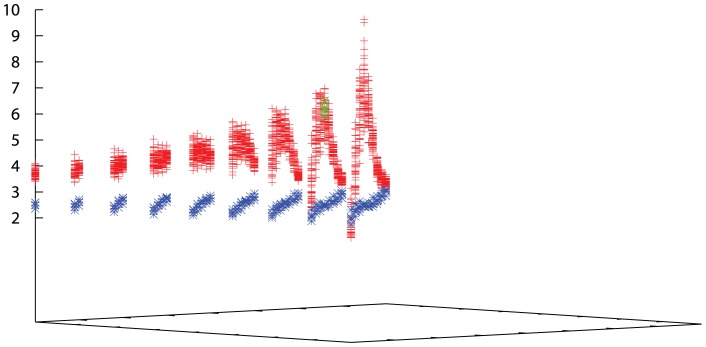
Score variation with peak width and displacement. Values of the peak-ratio score are plotted for displacements to the start and end points of the segment over which the peaks are summed (corresponding to variations of the light-blue bar in Figure 8d). Data are plotted for symmetry generated variations for each of the ten final frames for the Eph/ephrin data (red) and the cadherin data (blue). The three dimensional plot is viewed down diagonal lines of data that have the same peak width (increasing to the left). The green crosses mark the observed data at the “default” peak size of 10 microns used throughout. Variation in this region spans roughly 2 units of segregation score (5–7) which is 6+/−1 or 17% variation.

A related source of error in the calculation of the peak-ratio score is the choice of the range over which the peak heights are summed. It can be seen from Figures 7b and 8d that there is little scope for movement in the chosen range. Nevertheless, a range of displacements were tested for different peak widths and the maximum score obtained over each data set was found. For the cadherin cells the range of score was found to span almost the exact range as the variation over the data sets with the “default” peak width and position, indicating that, in each data set, this was either optimal or insensitive to small variations. (Figure 11, blue points). For the Eph/ephrin data, the corresponding data show greater variation (Figure 11, red points), especially when the peak width is small. Considering only the peak width of 10 microns used above, an estimate of between 15–20% variation is possible over the combined data from the last ten frames and the symmetry variations.

Other less well-defined sources of error on the theoretical side include the estimation of the cell density at which the MSD data were gathered and the extent to which the cells might have aggregated before the measurements were made. Taking an extreme position and measuring the MSD for the simulated cadherins at their full density (950 cells/field) and at the end of their simulation (4000 steps) reduced the slope of their MSD plot and hence the conversion factor *F* by half, bring the estimated real time close to the observed. This test sets a lower bound to the contribution, but it is unlikely that either of these extremes were approached, with a 10–20% reduction in time being a more reasonable estimate of the size of error that might be attributed to these effects.

#### 2.2.4 Eph/ephrin segregation over longer times

**Figure 12:**
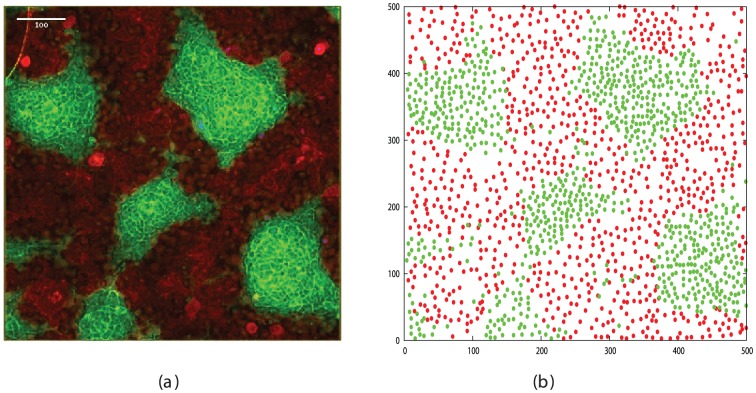
Eph/ephrin cell segregation after two days. Part *a* shows an image of the cells at the end of the experiment and part *b* shows the corresponding automatically digitised data derived from the image.

**Figure 13:**
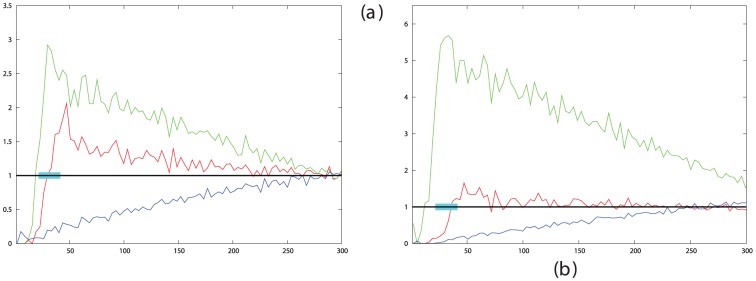
RDF plot for long Eph/ephrin segregation taken from the images obtained from two experiments in which the cells were allowed to segregate for two days. Plot *a* was calculated from the image and data shown in Figure 12. The configuration of cells giving rise to plot *b* had separated into two green clusters giving rise to the more extreme values (see legend to Figure 5 for an explanation of the plots and note the change in Y-axis scale in plot *b*). In both plots it can be seen that the separation of the red cells has increased, shifting the red peak in the plots away from the measured region (light-blue bar). This makes the value of the segregation score less reliable.

In order to obtain some data at more complete stages of segregation, the Eph/ephrin cells were allowed to segregate for as long as the cadherin cells, over a period of two days. Two experiments were performed with the cells attaining a degree of segregation with peak-ratio scores of 21 and 95, although these values must be treated with caution as it is apparent from Figure 12 and the corresponding RDF plots (Figure 13) that the behaviour of the two cell types has diverged, with a greater spacing being seen between ephrinB1 cells. The implications of this for the model will be reconsidered in the Discussion section, but at face value, these results are similar to the state of segregation found by simulation after roughly 100 K and 1 M cycles, respectively (Figure 2). With the conversion factor used above for the shorter Eph/ephrin experiment, this translates to a predicted time of 144 days compared to the two days actually taken, giving a comparable disparity in time to that seen in the shorter experiment.

### 2.3 Modifications to the adhesion model

To determine if it is possible to reduce the time required for segregation in our simulations, we turned to a closer examination of the underlying biological processes, beginning with the possibility that the generic “stickiness” of cells, mediated by the ubiquitous cadherin receptors, becomes stronger over the time that cells remain together. Such adhesion strengthening is known to occur [17], [18]. In the context of our model, this would still allow cells freedom of movement in the early stages of the partitioning process while allowing clusters of cells to become resistant to breaking apart in the later stages.

#### 2.3.1 Increasing cell adhesion with association time

The encoding of this behaviour into the model required the inclusion of a counter associated with each cell-cell cross-link which starts counting when a link is made and is reset to zero when it is broken. The value of this counter was then used to moderate the effect of the parameter unhook with a time scale determined by the value of the parameter sieze (Methods Sect. 4.1.2). The effect of sieze is simply to modify the value of unhook as a function of time using a Gaussian (half bell-shaped) function for which sieze determines the width. As the effects of sieze and unhook are closely linked, simulations were run over the full range of unhook values for different values of sieze.

**Figure 14:**
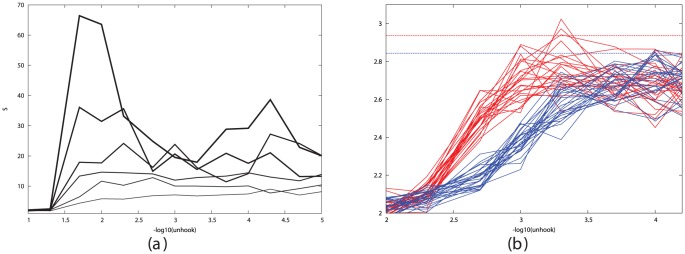
Clustering with increasing cross-link strength. *a*. The effect of increasing stickiness on clustering was plotted as in Figure 1 for simulations of different lengths from 10 K to 500 K, which appear as a series of traces with the longer runs in bolder lines. These results differ from those in Figure 1 by having the strength of links between cells increase with their linkage time, controlled by the parameter sieze = 100 (the results for unhook = 0 are not plotted in this graph). *b*. Using simulated cells at the same density as the cadherin cells and with a value of sieze = 600, 25 simulations were run for 4000 cycles over a range of unhook values (red lines) and compared to the results with no time dependent adhesion (blue lines), corresponding to a very large value of sieze. The dashed lines mark the mean of the top 20% of runs at the optimal value of unhook for the plots of corresponding colour.

The effect of increasing adhesion strength with the time cells are linked was to shift the transition point between clustering and non-clustering behaviour towards lower values of unhook, as this parameter now has an overall lower mean value. Besides this effect, the value of sieze made little difference to the extent of clustering observed. There was some marginally better clustering observed for sieze = 200 to 800 and a value of 600 was used to estimate the time needed to attain segregation for the cadherin experiment (Figure 14b). Although the increase in segregation score is small (0.1, at best 20%), with the low slope of the score with time for the cadherin data (Figure 10), this translates into a reduction of 500 steps in the time needed to attain the observed degree of segregation, an approx. 25% reduction.

#### 2.3.2 Increasing cell volume exclusion

The model of volume exclusion used in the simulations was stochastic, reproducing the observed behaviour in which cells often remain in close contact without adhesion or repulsion. Chance non-interacting close encounters between cells of like type will slightly increase the segregation score but as the like-type peaks are large in a segregated population, the effect remains small. However, in a well segregated population, the mixed-type peak will be small and chance close encounters may make a significant contribution. To investigate this, the repulsion of cells upon contact was increased.

The observed effect of this change was small for the cadherin cells, increasing the segregation score from 2.8 to 3.1, based on an average of ten runs with 950 cells for 4000 steps. A small change is not unexpected since the cells are dense, giving little scope to displace the mixed-type contacts without others being created. However, as was seen above, a small change in the segregation score can translate to a significant time shift and this change was estimated from Figure 10 to correspond to a reduction of 1000 time steps (50%), giving a real-time estimate of 5 days.

By contrast, the change to the Eph/ephrin cell segregation was more marked, increasing the segregation score from 6 to 9. This cell population is half as dense as the cadherin cells giving greater scope to create space between the mixed-type contacts. The corresponding time shift is difficult to estimate directly from the plots and required the simulations to be re-run over a more suitable time range.

#### 2.3.3 Combined effects

The two variations on the basic method evaluated above led to a 25% and 50% reduction in the estimated segregation time for the cadherin mediated cells and maybe much more for the Eph/ephrin mediated cells, but it is necessary to test whether these contributions are cumulative. With both changes implemented, a series of simulations were run again across a range of run-times in which the simulated segregation was likely to match the observed (as in Figure 10).

For the cadherins, the intersect now occurred at 2600 steps and for the Eph/ephrins at 6000, which converts into real times of 104 hours and 42 hours, respectively. The simulations of cadherin-mediated segregation now take twice as long as expected and the Eph/ephrin segregation eight times as long. Given the estimate of errors evaluated above, the simulations of differential adhesion may come within experimental error, but cannot account for segregation driven by Eph/ephrin interactions.

## Discussion

We have conducted experiments on two pairs of cell lines that exhibit segregation mediated by distinct sets of cell surface molecules: N-cadherin/E-cadherin and EphB2 receptor/ephrinB1. It is well established that differential adhesion mediated by cadherin expression can drive cell segregation [4], [3], [2], and recent studies have shown that Eph-ephrin interactions lead to decreased adhesion between cell populations due to cadherin cleavage [8]. The behaviour of these cells was compared to a simple mechanical model of cell segregation based on the preferred adhesion of cells of like type. Using the slopes of the mean square displacement (MSD) plot for the simulated and observed cells, we were able to determine how the simulation (virtual) time corresponded to the real time of the experiments.

We tested the effect of adjusting a number of parameters in the model to determine whether these affect the rate and extent of segregation. The degree of adhesiveness was found to be important, reflecting that with too low an adhesion like-cell clusters are not stable, whereas if adhesion is too strong the reorganisation of cells to find new neighbours is slower. Adjustments based on adhesion strengthening and cell volume exclusion made a smaller contribution to the segregation. There is also inherent error in the measurement of the degree of cell segregation and migration rate both in experiments and simulations, which might account for up to 50% variation in the time estimation. Taking these factors into account we conclude that a differential adhesion model can account for the behaviour of the cadherin mediated cell segregation results. However, for Eph/ephrin mediated segregation, which is much more extensive and rapid than that driven by N-cadherin/E-cadherin, even this margin of error still leaves a large absolute difference between predicted and observed times. This remaining disparity cannot be rectified by changing the the speed or “stickiness” of the cells in the simulation as any gain is renormalised by a change in the slope of the MSD plot.

An additional biological factor that was not part of the model is that during the experiment, the cells are still dividing. As cells give rise to daughters of like type and as the daughters can adhere and remain close, the overall effect of division is to increase segregation. With no easy way to automatically identify cell division, the scale of this effect can only be assessed indirectly from an increase in cell numbers over the simulation, which for the Eph/ephrin cell cultures was 10%. An equivalent contribution could be sufficient to account for the remaining gap between the simulated and observed times for the cadherin expressing cells. However, a similar contribution would not account for the difference in estimated time and observed time for the Eph/ephrin mediated segregation.

In conclusion, we have shown in this work that a simple model of cell adhesion, plus some minor adjustments and some allowance for cell division, is sufficient to account for the degree of cell segregation mediated by differential expression of cadherins. However, it is too slow, by roughly an order of magnitude, to account for the segregation mediated by interactions between Eph receptor and ephrin expressing cells. This implies that an alternative, or additional, mechanism is at work in these cells and it seems probable that this involves cell repulsion and migratory responses that occur upon Eph/ephrin interactions.

## Methods

### 4.1 A model for cell movement and adhesion

#### 4.1.1 Basic mechanical model

Previously, we have described how cells can be modelled by a ring of ten points with a central point representing the approximate centroid of the ring [13]. Each point is able to move, with the structure of the cell maintained by constraints imposed between the points. On each step (or frame) of the simulation, all points are displaced and a move of the central point is also applied to each point in the ring. Overall, this combination of random moves produces a Brownian-like motion of the cells. Superimposed over this, is a more directed motion that simulates the effect of an actin-based leading edge that can persist until another edge emerges in a new random direction.

Each point in the cell surface (referred to as “bodies” below) is also able to cross-link to bodies in other cells. These links persist until they are broken after a random time period controlled by an exponential decay rate. The value of the parameter (unhook) that sets this rate determines how “sticky” the cells are. A value of unhook = 0 means that the links have no chance to break whereas unhook = 1 means that they are immediately broken. Previous studies have found that unhook values in the range of to gives rise to a degree of adhesion that is typical for *in vitro* cell cultures [13].

#### 4.1.2 Increasing cell adhesion with time

A counter was associated with each of the ten bodies in the cell and initialised to zero at the start of the simulation. The counter was incremented with every time step that the body was cross-linked to another cell and when the link was broken, the counter was reset to zero.

The value of the counter was used to moderate the effect of the parameter unhook (above), using the following Gaussian relationship:
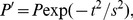
(1)where *P* is the value specified by unhook, *t* is the number of time steps counted since the link was formed and *s* sets the drop-off with time (equivalent to the standard-deviation of the normal distribution). The equation results in the modified chance to unhook, *P′* which ranges from the original value of unhook (*P*) at the start of the linkage to almost zero (a permanent link) over a long period. The length of this period is determined by *s* and for times after about 4 to 5 *s*, the link has effectively become permanent. The value of *s* was specified by a new parameter in the model called: seize which should adopt a value that gives easier movement in the early portion of a simulation (say, over the first 1000 time steps) then leads to increasing linkage strength. Values of seize from 10 to 1000 were tested.

### 4.2 Measuring cell segregation

**Figure 15:**
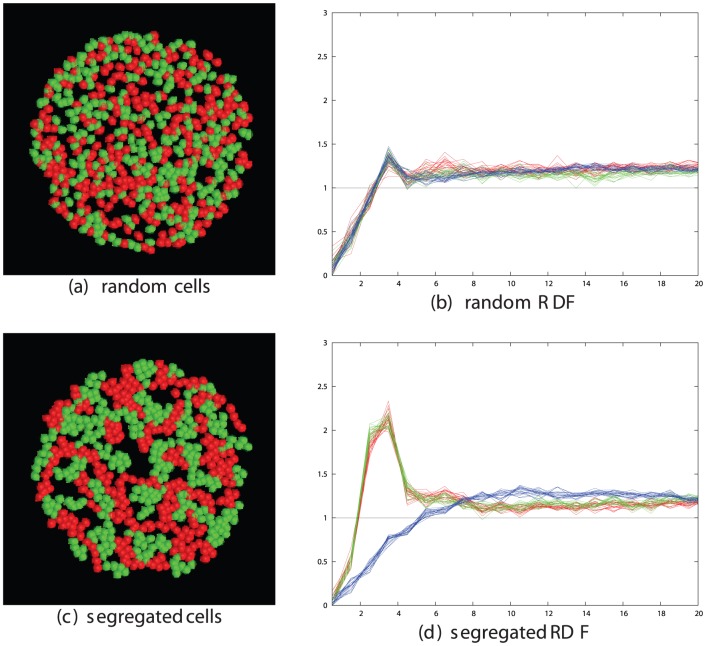
Example simulations and their RDF plots. Panels on the left show the configurarion of 600 cells of which half are red and half green after a simulation of 10,000 steps. Panel *a* has a random mix of cell positions whereas in panel *c*, significant clustering of like-type has occurred. Panels on the right plot the radial distribution function of the cells, which is the inormalised frequency of cell occurrence with increasing cell-cell separation. This is plotted separately for red pairs (red lines), green pairs (green lines) and red/green pairs (blue lines) for 10 frames at the end of the simulation. The expected random frequency is 1 but when segregation is present, the number of close contacts of like type is more than chance and the number of mixed contacts is less. These changes were built into a score **S** (Equation 2) to quantify the degree of segregation.

We used the Radial Distribution Function (RDF) to quantify cell sorting for varying cell densities in a field of cells, half of which were of “red” type (R) and half “green” (G). Cell segregation was measured by plotting separate RDFs for each cell population of like type and a third RDF for cells of unlike type. Clustering can then be identified as high peaks at the linkage separation in the RDF for like types and low for unlike. Peak areas were taken as the sum over three bins, and to avoid the calculation of peak heights being based on a single time point, 20 samples from equidistant points across the final 1000 frames of the simulation were used to obtain an average value. (Figure 15). These values were all combined in a single segregation score, *S*, as:
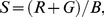
(2)where *R* and *G* are the summed peaks for the red and green cells, respectively, and *B* is the sum over the mixed interactions.

**Figure 16:**
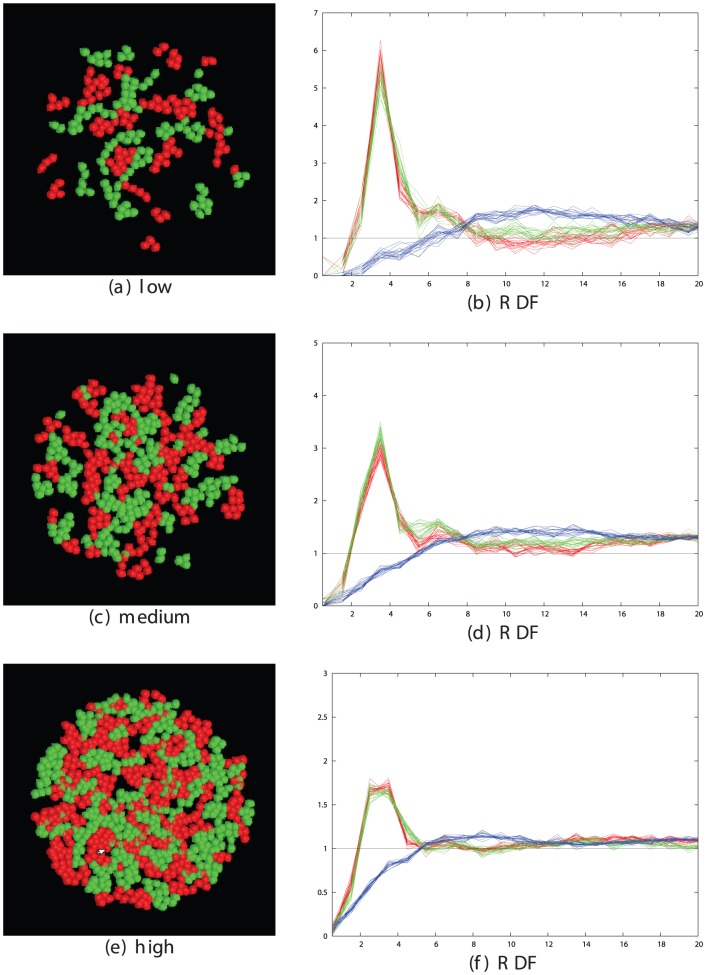
RDF peak changes with cell density. The size of the cell contact peaks in the RDF plot depends on the density of the cells. To illustrate this, sets of data are shown for three different cell densities: low (top) with 200 cells/field, medium (middle) with 400 cells/field and high (lower) with 800 cells/field. All are plotted as described in Figure 15. The size of the mixed type peak remains relatively constant at 0.5 but the red and green peaks drop by almost a factor of two from high to medium and medium to low density, with a corresponding drop in the segregation score (Equation 2). Note that there is a change in the Y-axis scale between plots.

At low density (100 cells in the field) linked cells in close proximity are a relatively unexpected event and consequently result in a high peak in the RDF, typically an order-of-magnitude over what is expected by chance. With increasing cell density, the random expectation for adjacent cells rises and the peak at the linkage separation correspondingly drops, attaining only a factor of 3 over background at densities when roughly half the surface area is covered with cells (500 cells). Close to the limit of the number of cells in the field (800–1000 cells), there are many enforced neighbours, irrespective of their linkage state and the typical peak height is reduced to under 2 (Figure 16).

### 4.3 Cell lines

L cells and L cells expressing mouse E-cadherin were obtained from Yasuyuki Fujita (Hokkaido University, Japan) [19] and L cells expressing mouse N-cadherin in a pCDNA3.1/myc-His vector were generated by Nobue Itasaki (University College Dublin, Ireland) using the same protocol. The EphB2/ephrinB1 cells were described previously [10].

#### 4.3.1 Cell sorting assay

**Figure 17:**
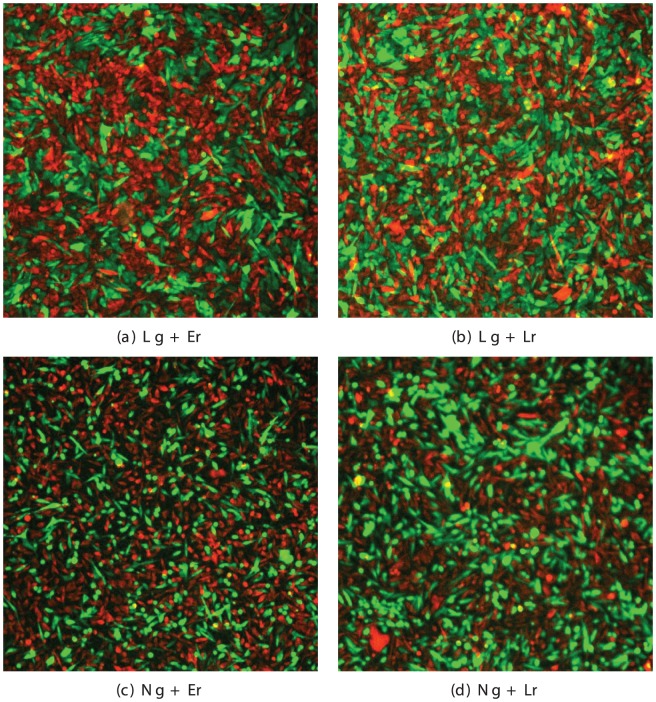
Cadherin-mediated cell segregation. Four combinations of L-cell lines are shown: *a)* L-cells+L-cells(E-cad), *b)* L-cells+L-cells, *c)* L-cells(N-cad)+L-cells(E-cad) and *d)* L-cells(N-cad)+L-cells, with the cell type named in the order green+red and the expression of each cadherin type (N and E) indicated in parentheses.

Wild-type L-cells, L-cells expressing E- and N-cadherin, HEK293 cells, HEK293 cells expressing mouse EphB2-receptor and mouse ephrinB1 were cultured at 37C with 5% CO, in DMEM supplemented with 10% FCS, glutamine and antibiotics. Before the experiment, cells were labeled with CMFDA (green) or CMRA (red) cell tracker dyes (Invitrogen) and dissociated with Accutase (PAA Laboratories). After washing with culture medium two differently labeled cells lines were mixed in equal proportions and plated onto a fibronectin-coated coverglass system (chambered 1.0 borosilicate; Lab-Tek) at a total density of 100,000–150,000 cells/cm^2^. Cells were then incubated at 37C for 24–72 hrs and visualized using an RT live-imaging workstation (Deltavision; Applied Precision, LLC) on a microscope (IX-70; Olympus) with a 10×/0.4NA objective (Olympus). Images of cells were processed using ImageJ image-processing program and coordinates of cell centres were identified manually using the ImageJ Cell counter plugin. The typical cell sorting pattern in mixtures of L-cells after 48 hrs in co-culture is presented in Figure 17.

#### 4.3.2 Western Blotting assay

**Figure 18:**
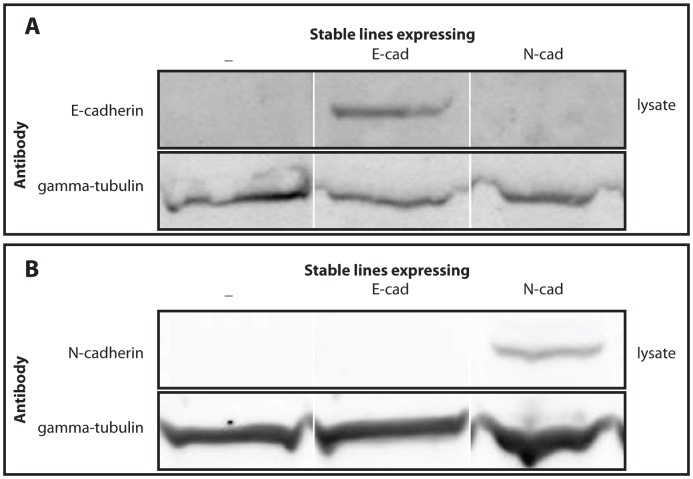
Expression of E- and N-cadherin in stable L-cell lines. Western blots of cell lysates stained with anti-E-cadherin antibodies (A) and anti-N-cadherin antibodies (B). Anti-gamma-tubulin staining (A, B) was used as a loading control.

Cell lysis and subsequent Western blotting was performed using a previously published protocol [16]. 30 micro-g of protein per condition was loaded onto a NuPAGE 10% bis/Tris gel (Invitrogen) and transferred to Immobilon FL membranes (Millipore). The membranes were stained with mouse anti-N-cadherin (BD Biosciences, Cat. 610920), mouse anti-E-cadherin antibodies (BD Biosciences, Cat. 610181), and rabbit anti-gamma-tubulin antibodies (Sigma, Cat. T3559) used as loading control. The membranes were then stained with secondary antibodies conjugated to infrared fluorescent dyes IR700 and IR800 (Rockland Immunologicals) and scanned using an infrared imager (Odyssey; Li-COR Biosciences). The results of Western blotting of L-cell lysates using anti-E-cadherin and anti-N-cadherin antibodies are presented in Figure 18.

## References

[1] Townes PL, Holtfreter J (1955) Directed movements and selective adhesion of embryonic amphibian cells. J Exp Zool 128: 53–120. 10.1002/jez.a.114 15559931

[2] Duguay D, Foty RA, Steinberg MS (2003) Cadherin- mediated cell adhesion and tissue segregation: qualitative and quantitative determinants. Dev Biol 253: 309–323. 12645933 10.1016/s0012-1606(02)00016-7

[3] Foty RA, Steinberg MS (2005) The differential adhesion hypothesis: a direct evaluation. Dev Biol 278: 255–263. 15649477 10.1016/j.ydbio.2004.11.012

[4] Steinberg MS, Takeichi M (1994) Experimental specification of cell sorting, tissue spreading, and specific spatial patterning by quantitative differences in cadherin expression. Proc Natl Acad Sci U S A 91: 206–209. 8278366 10.1073/pnas.91.1.206 PMC42915

[5] Steinberg MS (1970) Does differential adhesion govern self-assembly processes in histogenesis? equilibrium configurations and the emergence of a hierarchy among populations of embryonic cells. J Exp Zool 173: 395–433. 5429514 10.1002/jez.1401730406

[6] MS Steinberg (2007) Differential adhesion in morphogenesis: a modern view. Curr Opin Genet Dev 17: 281–286. 17624758 10.1016/j.gde.2007.05.002

[7] Dahmann C, Oates AC, Brand M (2011) Boundary formation and maintenance in tissue development. Nat Rev Genet 12: 43–55. 21164524 10.1038/nrg2902

[8] Solanas G, Cortina C, Sevillano M, Batlle E (2011) Cleavage of E-cadherin by ADAM10 mediates epithelial cell sorting downstream of EphB signalling. Nat Cell Biol 13 9: 1100–1107. 21804545 10.1038/ncb2298

[9] Batlle E, Wilkinson DG (2012) Molecular mechanisms of cell segregation and boundary formation in development and tumorigenesis. Cold Spring Harb Perspect Biol 4 1: a008227 doi: 10.1101/cshperspect.a008227. 22214769 10.1101/cshperspect.a008227 PMC3249626

[10] Poliakov A, Cotrina M, Wilkinson DG (2004) Diverse roles of eph receptors and ephrins in the regulation of cell migration and tissue assembly. Dev Cell 7: 465–480. 15469835 10.1016/j.devcel.2004.09.006

[11] Noren NK, Pasquale EB (2004) Eph receptor-ephrin bidirectional signals that target Ras and Rho proteins. Cell Signal 16: 655–666. 15093606 10.1016/j.cellsig.2003.10.006

[12] Graner F, Glazier JA (1992) Simulation of biological cell sorting using a two-dimensional extended Potts model. Phys Rev Lett 69: 2013–2016. 10046374 10.1103/PhysRevLett.69.2013

[13] Taylor WR, Katsimitsoulia Z, Poliakov A (2011) Simulation of cell movement and interaction. J Bioinfo Compu Biol 9: 91–110. 10.1142/s0219720011005318 21328708

[14] Niessen CM, Gumbiner BM (2002) Cadherin-mediated cell sorting not determined by binding or adhesion specificity. J Cell Biol 156: 389–399. 11790800 10.1083/jcb.200108040 PMC2199232

[15] Shan WS, Tanaka H, Phillips GR, Arndt K, Yoshida M, et al (2000) Functional cis-heterodimers of N- and R-cadherins. J Cell Biol 148: 579–590. 10662782 10.1083/jcb.148.3.579 PMC2174798

[16] Poliakov A, Cotrina ML, Pasini A, Wilkinson DG (2008) Regulation of EphB2 activation and cell repulsion by feedback control of the MAPK pathway. J Cell. Biol 183: 933–947 2008. 19047466 10.1083/jcb.200807151 PMC2592822

[17] Ehrlich JS, Hansen MD, Nelson WJ (2002) Spatio-temporal regulation of Rac1 localization and lamellipodia dynamics during epithelial cell-cell adhesion. Dev Cell 3: 259–270. 12194856 10.1016/s1534-5807(02)00216-2 PMC3369831

[18] Yamada S, Nelson WJ (2007) Localized zones of Rho and Rac activities drive initiation and expansion of epithelial cell-cell adhesion. J Cell Biol 178: 517–527. 17646397 10.1083/jcb.200701058 PMC2064836

[19] Dupre-Crochet S, Figueroa A, Hogan C, Ferber EC, Bialucha CU, et al (2007) Casein kinase 1 is a novel negative regulator of E-cadherin-based cell-cell contacts. Mol Cell Biol 27: 3804–16 Epub. 17353278 10.1128/MCB.01590-06 PMC1899980

